# Addition of Dairy Lipids and Probiotic *Lactobacillus fermentum* in Infant Formulas Modulates Proteolysis and Lipolysis With Moderate Consequences on Gut Physiology and Metabolism in Yucatan Piglets

**DOI:** 10.3389/fnut.2021.615248

**Published:** 2021-02-24

**Authors:** Marion Lemaire, Olivia Ménard, Armelle Cahu, Isabelle Nogret, Valérie Briard-Bion, Benoit Cudennec, Isabelle Cuinet, Pascale Le Ruyet, Charlotte Baudry, Didier Dupont, Sophie Blat, Amélie Deglaire, Isabelle Le Huërou-Luron

**Affiliations:** ^1^Institut NuMeCan, INRAE, INSERM, Univ Rennes, St-Gilles, France; ^2^Lactalis R&D, Retiers, France; ^3^STLO, INRAE, Institut Agro, Rennes, France; ^4^UMR Transfrontalière BioEcoAgro, Univ. Lille, INRAE, Univ. Liège, UPJV, YNCREA, Univ. Artois, Univ. Littoral Côte d'Opale, ICV – Institut Charles Viollette, Lille, France

**Keywords:** infant formula, milk fat, probiotic, digestion, intestinal physiology, glucagon-like peptide-1

## Abstract

Breast milk is the gold standard in neonatal nutrition, but most infants are fed infant formulas in which lipids are usually of plant origin. The addition of dairy lipids and/or milk fat globule membrane extracts in formulas improves their composition with beneficial consequences on protein and lipid digestion. The probiotic *Lactobacillus fermentum* (Lf) was reported to reduce transit time in rat pups, which may also improve digestion. This study aimed to investigate the effects of the addition of dairy lipids in formulas, with or without Lf, on protein and lipid digestion and on gut physiology and metabolism. Piglets were suckled from postnatal days 2 to 28, with formulas containing either plant lipids (PL), a half-half mixture of plant and dairy lipids (DL), or this mixture supplemented with Lf (DL+Lf). At day 28, piglets were euthanized 90 min after their last feeding. Microstructure of digesta did not differ among formulas. Gastric proteolysis was increased (*P* < 0.01) in DL and DL+Lf (21.9 ± 2.1 and 22.6 ± 1.3%, respectively) compared with PL (17.3 ± 0.6%) and the residual proportion of gastric intact caseins decreased (*p* < 0.01) in DL+Lf (5.4 ± 2.5%) compared with PL and DL (10.6 ± 3.1% and 21.8 ± 6.8%, respectively). Peptide diversity in ileum and colon digesta was lower in PL compared to DL and DL+Lf. DL and DL+Lf displayed an increased (*p* < 0.01) proportion of diacylglycerol/cholesterol in jejunum and ileum digesta compared to PL and tended (*p* = 0.07) to have lower triglyceride/total lipid ratio in ileum DL+Lf (0.019 ± 0.003) as compared to PL (0.045 ± 0.011). The percentage of endocrine tissue and the number of islets in the pancreas were decreased (*p* < 0.05) in DL+Lf compared with DL. DL+Lf displayed a beneficial effect on host defenses [increased goblet cell density in jejunum (*p* < 0.05)] and a trophic effect [increased duodenal (*p* = 0.09) and jejunal (*p* < 0.05) weights]. Altogether, our results demonstrate that the addition of dairy lipids and probiotic Lf in infant formula modulated protein and lipid digestion, with consequences on lipid profile and with beneficial, although moderate, physiological effects.

## Introduction

Early nutrition is essential to ensure optimal infant growth and development, especially regarding the digestive functions, which are immature at birth (1). While human milk is recognized as the gold standard for infant nutrition, a large proportion of them are formula-fed (2). Infant formulas have been much improved over the last decades regarding their nutritional content; however, differences remain between infant formulas and human milk regarding their non-nutritive composition, i.e., protein/lipid structure, oligosaccharide, and bacterial content (3).

At the supramolecular level, human and bovine milk fat is organized under its native form into dispersed globules enveloped in a biological membrane called *milk fat globule membrane* (MFGM). However, the step of homogenization during infant formula processing is conducted to uniformly distribute submicronic droplets and impact the compositional and organizational architecture of the lipid–water interface (3).

At the molecular level, the triacylglycerol structure, i.e., the regiodistribution of the fatty acids, impacts fatty acid absorption. This is of particular importance for palmitic acid, a major fatty acid in human milk. In the latter, more than 70% of the palmitic acid is located in the sn-2 position, while this is true for <20% of the palmitic acid from palm oil, a major fat source for infant formula based on plant oil. Dairy lipids present an intermediate profile, with 40–45% of palmitic acid in this inner position (4, 5). Human pancreatic lipase is sn-1,3 specific, and when palmitic acid is located at these positions, its absorption as non-esterified fatty acids is reduced as it tends to form calcium soap that is excreted in stool. On the contrary, when palmitic acid is esterified in sn-2 position, pancreatic lipolysis results in the formation of water-soluble palmitoyl-monoglycerol, which is well-absorbed. Accordingly, palmitic acid and calcium have been shown to be better absorbed in breastfed infants as compared to infants fed plant oil–based formulas (6).

A better understanding of the impact of the formula matrix effect onto its digestion in the infant gastrointestinal tract is therefore essential to optimize their formulation and improve the health of formula-fed infants. However, ethical and financial constraints of clinical studies limit knowledge. *In vitro* systems designed to mimic infant digestion, although relevant, cannot reproduce all the biological complexity of the digestive tract, such as digestion and absorption, hormonal feedbacks, neural interactions, host and microbe interactions, and microbe and microbe interactions. Piglet was identified as a more appropriate *in vivo* model to study infant digestion (7). In this model, the addition of DLs and MFGM extracts was shown to have structural and biochemical consequences on infant formula digestion, decreasing small intestinal digestion of casein and β-lactoglobulin and leading to more numerous β-casein peptides in intestinal contents, but increasing small intestinal lipid digestion (8). Besides, the administration of MFGM extracts with probiotic *Lactobacillus fermentum CECT 5716* (Lf), lactic acid bacteria originally isolated from human milk (9), was shown to decrease the whole gut transit time of rat pups (10). Interestingly, this effect was not observed when MFGM extracts were provided alone, suggesting a potential role of probiotic Lf on infant formula digestion. Whether all these features remain true when DLs without additional supply of MFGM are provided alone or with the Lf probiotic in infant formulas is unknown.

It was previously demonstrated that the addition of dairy lipids to replace partially plant lipids and without additional supply of MFGM extracts and probiotic Lf in infant formula had a beneficial impact on the intestinal endocrine function later in adulthood, enhancing cecal GLP-1 content and GLP-1 meal-stimulated secretion in adult mini-pigs (11). However, the impact of infant formula containing dairy lipids with or without probiotic Lf on the piglet intestinal endocrine function and the consequences on pancreas maturation and glucose homeostasis are currently unknown. Addition of dairy lipids in the infant formula, inducing modification in proteolysis and the presence of peptides more distally in the intestine as described above, could stimulate GLP-1 secretion by the distal L-cells in ileum (12, 13), an effect that could be enhanced if transit time is also decreased as described above with Lf.

Our hypothesis was that the addition of dairy lipids and probiotic Lf in infant formulas could impact their digestion and consequently piglet lipid and protein metabolism. The objective of this study was to compare the digestion of infant formulas containing dairy lipids in the presence or absence of probiotic Lf to a reference formula containing only plant lipids and to evaluate their metabolic impact in infant formula-fed piglets.

## Methods

### Ethical Approval

The present study was designed and conducted in compliance with the current ethical standards of the European and French guidelines (directive 2010/63/EU and decree 2013-118, respectively). The ethics committees of CREEA (Rennes Committee of Ethics in Animal Experimentation) and of France's Ministry of Higher Education and Research approved the protocol (authorization #2016011111546978). Animals were observed daily throughout the experimental protocol to ensure their welfare, and they received no medication or antibiotic treatment.

### Animals and Study Design

The study design has already been published in Lemaire et al. (11). A total of 27 female and male (11 and 16, respectively) Yucatan piglets (Saint-Gilles, France) were used in three replicates. One animal was excluded from the analyses because of health issues. Piglets were separated from their sow at postnatal day (PND) 2 and housed in individual stainless-steel metabolic cages. They were fed one of the three experimental formulas with an automatic milk feeder as previously described (14) until weaning, i.e., at PND28. To account for litter-to-litter variation, three piglets with a body weight (BW) close to the mean birth weight of the litter were selected from each litter and assigned to one of the three formulas. Allocation to formulas was balanced between groups for birth weight, BW at PND2, and sex. Formulas were rehydrated each day at 20% dry matter extract in water before distribution. The formula was allocated in 10 meals automatically distributed during the day. BW was measured twice a week, and feeding amounts were adjusted accordingly. The daily net energy offered was 1450 kJ/kg BW^0.75^. Formula intake was automatically recorded for each meal. The average daily volume of formula intake was 220 ± 4 mL/kg (BW)^0.75^. Piglets were euthanized at PND28, and tissues were collected and weighed.

### Diets

Formulas were manufactured by Lactalis (Retiers, France) and adapted to meet piglet energy and protein requirements. The three formulas had the same energy, protein, lipid, and carbohydrate levels. They differed by the lipid origin, only plant lipids (*n* = 9) vs. half-half plant lipids and dairy lipids (*n* = 9), and the supplementation with Lf (DL+Lf, *n* = 8), as described in Table 1. Dairy lipids came from the cream, which may contain some residual MFGM that may account for 2–6% of the fat mass (15), but there was no addition of specific MFGM. The experimental formulas contained higher amounts of protein and lipid and a lower amount of lactose than a standard human infant formula in order to meet the piglet requirement. Lipid:protein and linoleic acid:α-linolenic acid (ω6:ω3 = 6–7) ratios were kept similar to those found in commercial infant formulas. The formulas were based on a mixture of skim milk and whey protein concentrate powders to reach a casein:whey proteins ratio of 30:70 wt/wt.

**Table 1:** Composition of infant formulas.

**g/100 g of powder**	**PL^a^**	**DL^a^**	**DL+Lf^a^**
Proteins	17.8	17.9	17.9
Lipids	43.6	44.7	44.6
Carbohydrates	33.1	32.3	32.2
Minerals	3.5	3.4	3.4
Energy (kJ)	2476	2506	2501
*Lactobacillus fermentum CECT 5716* (Lf)	—	—	1.9E+08
Phospholipids*^b^*	0.40	2.52	2.52
Cholesterol*^c^*	0.004	0.054	0.054

a *Formulas contained as lipids either only plant lipids (PL), a half-half mixture of plant and dairy lipids (DL), or a half-half mixture of plant and dairy lipids supplemented with Lf (DL+Lf). Formulas were rehydrated at 20% of dry extract. Lipid sources of the PL formula were palm oil (71.7%), rapeseed oil (23.2%), and refined sunflower oil (5.1%); those of the DL and DL+Lf formulas were cream (53.4%), rapeseed oil (21.1%), refined sunflower oil (13.1%), and high oleic sunflower oil (12.4%)*.

b *Concentrations of phospholipids hereby indicated were values obtained in a previous production batch of similar infant formulas*.

c *Calculated concentrations of cholesterol were based on the cholesterol content of ingredients used in PL and D L(±Lf) formulas*.

### Postmortem Sampling

According to our previous study on the kinetics of protein digestion, the 90-min postprandial sampling time is the optimal timepoint considering the progress of the digestion process and the appearance of dietary peptides in all digestive compartments from stomach to the ileum (16). Therefore, piglets were euthanized 90 min postprandially in the experimental slaughterhouse by electrical stunning immediately followed by exsanguination.

Blood was collected in tubes containing K^2^-EDTA for glucose, insulin, haptoglobin, and lipid profiles and in tubes containing K^2^-EDTA plus an anti–dipeptidyl peptidase-IV (DPP-IV, 10 μL/mL of blood) for GLP-1 (Millipore, Billerica, MA, USA). After centrifugation (10 min, 2,500 g, 4°C), plasma samples for glucose, insulin, haptoglobin, and lipid assays were stored at −20°C and the ones for GLP-1 assay at −80°C. Brain, liver, pancreas, perirenal, and subcutaneous adipose tissues were weighed. Duodenum, proximal and median jejunum, ileum, cecum, and colon were weighed (full and empty), and their length recorded. The digestive contents from the stomach, duodenum, proximal and median jejunum, ileum, and colon were collected by exerting a gentle pressure with the fingers.

Samples of digesta collected for *in vitro* intestinal secreting tumor cell line (STC-1) assays were stored at −20°C until used. Protease inhibitors were added in digesta collected for protein digestion analysis: pepstatin A (0.73 mM; 10 μL/mL of stomach digesta) and Pefabloc (0.1 M; 50 μL/mL of small and large intestinal digesta). Before storage at −20°C until further analysis, pH was measured. Digestive content aliquots were submitted to the direct lipid Folch extraction, as previously described (17). Briefly, 400 μL of digesta sample was mixed with 2.4 mL of chloroform/methanol (2:1 vol/vol) and acidified with 160 μL of HCl 0.1 N to stop lipolysis. The extract was then rinsed with NaCl 0.73% (100 μL) and 600 μL of chloroform/methanol (2:1 vol/vol). The chloroformic phase containing the lipid fraction was recovered and stored at −20°C for further lipid analyses. In addition, samples of the experimental formulas and digestive contents were collected for chemical and structural characterizations.

Proximal jejunal, ileal, and cecal tissue segments were rinsed with cold phosphate-buffered saline (PBS) and fixed in 4% paraformaldehyde for 48 h until further dehydration in ethanol and embedding in paraffin for morphometry analysis. Adjacent pieces of cecal and colonic tissues were rinsed with cold PBS and stored at −80°C until GLP-1 extraction and assay. Mucosa was scrapped from a 10-cm ileal segment for GLP-1 assays. One-cm^3^ sample of pancreas was directly frozen and stored at −80°C for insulin extraction and analysis, and another cm^3^ was fixed in 4% paraformaldehyde for immunohistochemistry.

### Structural Characterization of Experimental Formulas and Digestive Contents

#### Particle Size Measurements

Particle size distribution was measured on the experimental formulas by laser light scattering using a Mastersizer 2000 (Malvern Instruments, Malvern, UK), with two laser sources at 466 and 633 nm. Refractive indexes used were 1.462 for vegetable oil and 1.333 for water (dispersion solution in the measurement cell). Samples were diluted in MilliQ-water in the measurement cell, either directly or after a 10-fold dilution in sodium dodecyl sulfate (SDS 1%), an anionic surfactant allowing aggregate dissociation. Measurements were performed in triplicates. Mean particle size distribution was represented by distribution curve, and mode diameter (diameter of the most frequent particles), volume weighted mean diameter (D[4,3]), and the specific surface area (SS) developed by the particles were calculated.

#### Confocal Laser Scanning Microscopy

The microstructures of the experimental formulas and of the digestive contents of all tissue segments of the intestine were observed as previously described in Bourlieu et al. (17). Briefly, a Nikon C1Si confocal laser scanning microscopy was used on an inverted microscope TE2000-E (Nikon, Champigny-sur-Marne, France) operated with an argon laser (excitation at 488 nm) and two He-Ne lasers (excitations at 543 and 633 nm). A 40× oil-immersion objective was used for all images, and three fluorescent dyes were used to localize apolar lipids (Lipidtox®), proteins (Fast Green), and amphiphilic compounds (Rhodamine-DOPE®). The microstructure was assessed on the three infant formulas and on the digesta from three pigs per formula group (one per replicate).

### Biochemical Characterization

#### Proteins

SDS–polyacrylamide gel electrophoresis (PAGE) was performed on the experimental formulas and gastric contents using 4–12% polyacrylamide NuPAGE Novex *bis*-Tris 15-well-precast gels (Invitrogen, Carlsbad, CA, USA) and according to the manufacturer's instructions. Samples were diluted with NuPAGE® LDS sample buffer and then treated with 0.5 M dl-dithiothreitol and deionized water. The dilution of the formula by the gastric secretion was estimated based on the dry matter extract of the digesta compared to the dry matter of the formula. Twenty microliters of diluted sample containing 10 μg of protein was loaded into each well. Mark 12 Unstained Standard (Invitrogen) was used as a molecular weight (Mw) marker to identify the position of the bands. Gels were fixed in 40% (vol/vol) ethanol, 10% (vol/vol) acetic acid and 50% (vol/vol) deionized water and were rinsed for 30 min in deionized water before overnight staining with Bio-Safe Coomassie stain (Bio-Rad Laboratories, France). Discoloration of gels was performed with water. Image analysis of SDS-PAGE gels was carried out using Image Scanner III (GE Healthcare Europe GmbH, Velizy-Villacoublay, France), followed by densitometry of each band using the software Image Quant TL™ (GE Healthcare Europe GmbH, Velizy-Villacoublay, France). The percentage of each intact protein present in stomach 90 min postprandially was thus estimated in comparison with that present in the experimental formula.

#### NH_2_ Quantification

The method was adapted from Darrouzet-Nardi et al. (18). It consisted in a spectrophotometric microplate analysis based on the reaction of orthophthaldialdehyde with primary amines in the presence of dithiothreitol resulting in 1-alkylthio-2-alkylisondole detected at 340 nm. The total content of primary amines in the experimental formulas was determined after total acid hydrolysis (6N hydrochloric acid, at 110°C for 24 h in vacuum-sealed glass tubes).

#### Peptidomic Analysis

Peptides in the three infant formulas and in the digestive samples (from stomach to colon, *n* = 99) were identified and quantified semiquantitatively by tandem mass spectrometry (Q-exactive, Thermo Scientific, Sans Jose, USA), such as described in Deglaire et al. (19). A home-made database for bovine milk proteins, based on the protein sequences as reviewed in uniport.org, was used for peptide identification. Bioactive peptides were searched within the database of BIOPEP (20), and prediction of antimicrobial activity was examined using CAMPR3 (21). Peptides were considered as antimicrobial peptide (AMP) when the score reached at least 0.5 on both methods that were support vector machine and random forest classifiers, recognized as the best-performing AMP predictors (22).

### Lipid Analysis

#### Total Fatty Acids

The fatty acid composition of the formulas (C8:0–C24:0) was analyzed by gas chromatography coupled to a flame ionization detector by direct transmethylation, as described in Oliveira et al. (23). Samples were injected in duplicate.

#### Free Fatty Acids

Gastric free fatty acids (FFAs) (C4:0 to C20:0) were analyzed on gastric digesta by gas chromatography after lipid extraction using the Folch method and followed by a solid phase extraction (24). Three internal standards (160 μL of C5, C11, and C17 at 0.5 mg/mL) were added to samples prior to extraction as described previously (25). Samples were injected in duplicate. The gastric lipolysis degree was determined based on the total amount of FFA related to the estimated amount of total fatty acids present in the digestive content, the latter being determined based on the formula dilution such as indicated by the dry matter extract of the digesta.

#### Lipid Class Analysis

Thin-layer chromatography (TLC) was conducted to follow the evolution of the different lipid classes between stomach, proximal jejunum, and ileum digestive contents at 90 min postprandially as compared to undigested formula. A digesta volume equivalent to 60 μg of experimental formula lipids was spotted on silica gel plates (10 × 20 cm, 0.25 mm, Si G60, Merck). An Automatic TLC Sampler III (CAMAG, Muttenz, Switzerland) was used. Plates were immersed in hexane/diethyl ether/acetic acid (70:30:2 v·v^−1^v^−1^) and then stained by immersion in copper sulfate II/orthophosphoric acid solution and heated (15 min, 150°C). Image analyses of the plates were performed as described for SDS-PAGE gels, allowing semiquantification of the different lipid classes, using the undigested formula as the reference.

### Morphometry and Immunohistochemical Analyses

Seven-μm sections were stained with hematoxylin and eosin and examined under a light microscope (Nikon Eclipse E400, Nikon Instruments, France) using image analysis software (NIS-Elements AR 3.0, Nikon Instruments) as described (26). Villus, crypt, and goblet cell numbers were measured in at least 15–20 well-oriented crypt-villus units per piglet.

Fresh cecum, colon, and pancreas were fixed in 4% paraformaldehyde for 48 h at room temperature. They were then placed at 4°C in PBS containing 30% sucrose and embedded in the Tissue-Tek Optimum Cutting Temperature compound (Sakura Finetek Europe B. V., Zoeterwoude, the Netherlands), frozen in isopentane, and sectioned using a cryostat-microtome. Immunohistochemical analysis of cecum, colon, and pancreas was processed as previously described (11) to determine the number of enteroendocrine (chromogranin A-labeled) cells and GLP-1–secreting cells per area of mucosa, and the percentage of endocrine tissue and the number and diameter of islets in the pancreas.

### Glucose, Lipid, Haptoglobin, Insulin, and GLP-1 Assays

Plasma glucose, FFA, triglycerides, total cholesterol, high-density lipoprotein cholesterol and haptoglobin were assessed by an automated spectrophotometric method (Konelab 20i, Thermo Fisher Scientific, Illkirsh, France) using specific commercial kits (Biomérieux, Bruz, France). The intra-assay coefficient of variation was <5%.

Insulin content was extracted from the pancreas by homogenization in 10 mL of an ethanol acid solution (75% absolute ethanol, 23.5% ultrapure H_2_O, 1.5% HCl 12N) (Polytron 3100, Kinematica, 25,000 rpm, 2 × 20 s). After an overnight storage at −20°C, samples were centrifuged (30 min, 190 g, 4°C), and supernatants stored at −20°C. Ethanol acid solution (10 mL) was added to pellets for a second extraction, stored overnight at −20°C, and centrifuged (30 min, 190 g, 4°C). Supernatants were collected and pooled to the ones from the first extraction and diluted in a PBS/bovine serum albumin solution (1:3,000), and pancreas insulin concentration and plasma insulin were measured by a radioimmunoassay method, using iodinated porcine insulin as a tracer (INSULIN-CT, Cisbio International, Gif sur Yvette, France). The intra-assay CV and interassay CV were 15 and 11%, respectively, for a concentration of 35 μIU/mL.

GLP-1 content was extracted from ileal mucosa, cecum, and colon by homogenization of 1 g of tissue in 5 mL of ethanol acid solution (1% HCl 12M, 74% absolute ethanol, 25% H_2_O) (Polytron 3100, Kinematica, 24,000 rpm, 2 × 20 s). After 24 h at 4°C, samples were centrifuged (20 min, 2,000g, 4°C), and supernatants diluted (1:1,000, 1:300, and 1:250 for ileal mucosa, cecum, and colon, respectively). Intestinal and plasma GLP-1 concentration was measured using a GLP-1 active enzyme-linked immunosorbent assay kit (Millipore), according to the manufacturer's instructions.

#### *In vitro* STC-1 Cell Assays

This assay aimed to evaluate *in vitro* the impact of jejunal and ileal contents on the GLP-1 secretion of STC-1 cells, a murine intestinal tumor cell line that possesses many features of native intestinal enteroendocrine cells. The intestinal STC-1 cell line was obtained from ATCC (ATCC®, CRL-3254™). The STC-1 cells were grown (37°C, 5% CO_2_ atmosphere) in Dulbecco modified Eagle medium (4.5 g/L glucose) supplemented with 10% inactivated fetal calf serum (FCS), 100 U/mL penicillin, 100 μg/mL streptomycin, and 2 mM glutamine. When reaching 80% confluence, cells were washed with PBS (pH 7.4), trypsinized, and seeded onto 24-well culture plates at a density of 40 × 10^3^ cells per well and cultivated until they reached 70%−80% confluence. Cells were washed twice with media without FCS. Jejunal and ileal digesta were diluted (1:16 vol/vol) in incubation buffer (4.5 mM KCl, 1.2 mM CaCl_2_, 1.2 mM MgCl_2_, 140 mM NaCl, and 20 mM HEPES-Tris, pH 7.4) and centrifuged (10 min, 2,000 g, 4°C). Cells were then incubated for 2 h (37°C, 5% CO_2_ atmosphere) with the digesta supernatants or with the incubation buffer (control wells). Finally, cell supernatants were collected, centrifuged (7 min, 2,000 g, 4°C), and kept at −20°C before GLP-1 radioimmunoassay measurement using an active GLP-1 kit (GLP1A-35HK, EMD Millipore, Billerica, MA, USA).

### Statistical Analysis

Statistical analyses were performed using the R software, version 3.0.3 (27). Normality was tested with Shapiro and Wilk test. For parameters with unequal variances between groups, Box–Cox transformations were used. Differences between groups were assessed using a two-way analysis of variance (ANOVA) (lm function) testing formula composition, replication, sex, and interactions between formula and sex, and between formula and replication, followed by Tukey *post hoc* test (Tukey honestly significant difference). For lipid class characterization across intestinal sites, data were Box–Cox transformed and subjected to ANOVA for repeated measures (lme function) including formula, intestinal site, replication, sex, and interactions between formula and all other factors, followed by Tukey *post hoc* test (lsmeans package). Sex effect and the diet × sex interaction were not significant unless otherwise mentioned. Data are presented as mean values with their standard error of the mean (SEM). Differences were considered significant at *p* < 0.05 and a trend at *p* < 0.1. The graphical representation of the position and abundance of the peptides on the parent protein was performed using an in-house R script.

## Results

### Effects of the Addition of Dairy Lipids and Probiotic Lf on Infant Formula and Digestive Content Structures

**Figure 1:**
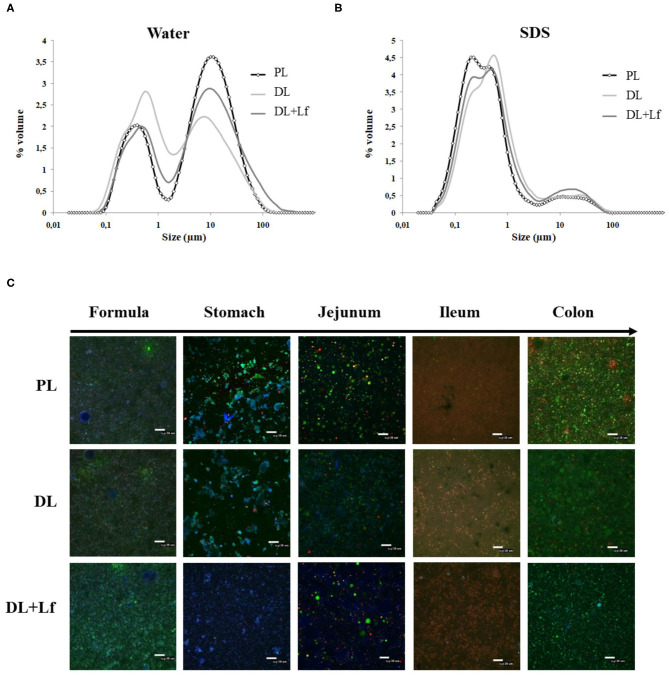
Structural characterization of PL, DL, and DL+Lf formulas. **(A,B)** Particle size distribution of undigested PL, DL, and DL+Lf formulas determined by laser light scattering in **(A)** water and **(B)** SDS. Data represent the mean of three samples, each of them being measured in triplicate. **(C)** Confocal laser scanning microscopy images (objective ×40 zoom 1) of PL, DL, and DL+Lf undigested formulas and digestive contents from stomach to colon. Apolar lipids are colored in green (Lipidtox®), amphiphilic compounds in red (Rhodamine-PE®), and proteins in blue (fast green). Scale bars = 30 μm. Formulas contained as lipids either only plant lipids (PL), a half-half mixture of plant and dairy lipids (DL), or a half-half mixture of plant and dairy lipids supplemented with Lf (DL+Lf) sulfate.

The three infant formulas were all characterized by a bimodal distribution of the particle size in water (Figure 1A, mode 1 = 9.77 μm and mode 2 = 0.52 μm). The biggest mode (mode 1) mostly disappeared after SDS addition (Figure 1B), indicating that these larger particles were aggregated droplets.

The microstructure of the digestive contents 90 min postprandially is given in Figure 1C. There was no major difference among formula groups within an intestinal site, whereas differences between intestinal sites were observed. The gastric phase was dominated by strong emulsion destabilization and protein aggregation. Lipid droplets were inserted within protein aggregates, which were likely formed due to the combined effect of acidic pH (3.4 in average at 90 min postprandially, Table 2) and proteolysis. The digesta structure in jejunum greatly differed from that in the stomach, with fewer protein aggregates likely due to the digestive process (hydrolysis, emptying, and dilution by endogenous fluids) combined with the pH increase (Table 2). Mixed droplets of apolar and amphiphilic compounds, created by the bile salt emulsification, were also observed in the jejunum. There was mainly a colocalization of apolar lipids and amphiphilic molecules in the ileum (Figure 1C). The digesta structure in the colon was different from that of the ileum, with great quantity of apolar lipids, possibly originating from undigested lipids of the infant formula.

**Table 2:** Digestive contents pH of PL, DL, and DL+Lf formulas.

**pH**	**PL**	**DL**	**DL+Lf**	**Diet effect** ***p* value**
Stomach	3.41 ± 0.30	3.37 ± 0.45	3.39 ± 0.30	0.98
Duodenum	4.64 ± 0.20	4.74 ± 0.30	4.80 ± 0.23	0.90
Proximal jejunum^*^	5.66 ± 0.15	5.42 ± 0.12	5.48 ± 0.13	0.17
Median jejunum	6.66 ± 0.10	6.90 ± 0.06	6.65 ± 0.14	0.20
Ileum	7.64 ± 0.11	7.72 ± 0.10	7.66 ± 0.15	0.46

*Data are expressed as the mean ± SEM*.

* *Proximal jejunum: p(sex) < 0.01 (females > males)*.

*Formulas contained as lipids either only plant lipids (PL, n = 5–8), a half-half mixture of plant and dairy lipids (DL, n = 5–7), or a half-half mixture of plant and dairy lipids supplemented with Lf (DL+Lf, n = 4–7)*.

Gastric pH and intestinal pH (Table 2) were not impacted by the formula composition, increasing from the stomach up to the ileum.

### Effects of the Addition of Dairy Lipids and Probiotic Lf on Piglet Growth, Body Composition, and Intestinal Morphology

BW gain, energy intake, and food efficiency were similar between groups during the lactation period (Supplementary Figures 1A–C). BW at euthanasia and tissue relative weights were similar between groups except for the liver (DL > PL), duodenum (tendency DL+Lf > DL), and the median jejunum (DL+Lf > PL and DL) (Supplementary Table 1). Total small intestinal length tended to be higher in DL+Lf compared with DL. Goblet cell number was higher in the proximal jejunum of DL+Lf than PL piglets, whereas villi surface tended to be lower in the ileum of DL+Lf than PL piglets (Supplementary Table 1); neither was different to DL.

### Effects of the Addition of Dairy Lipids and Probiotic Lf on Proteolysis

**Figure 2:**
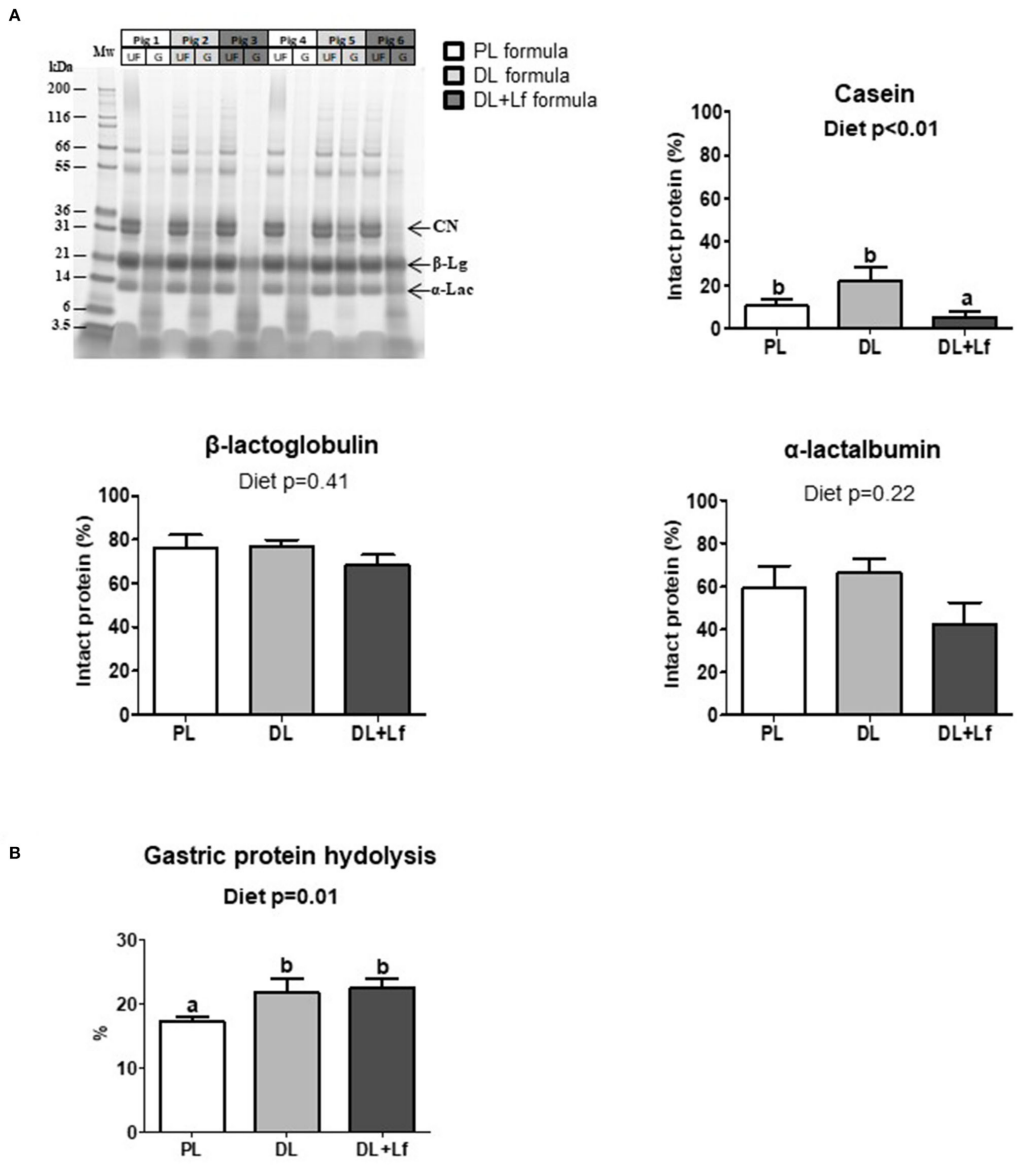
Gastric proteolysis of PL, DL, and DL+Lf formulas. **(A)** Example of SDS-PAGE protein profiles of PL, DL, and DL+Lf formulas during gastric *in vivo* digestion. Protein molecular mass standards (Mw) are on the left followed by the undigested infant formula (UF) and corresponding gastric content (G) for the same pig (side by side). Gastric proteolysis resistance: corresponding percentage of intact proteins [casein (CN), β-lactoglobulin (β-Lg), and α-lactalbumin (α-Lac)] during gastric digestion, estimated by densitometry in comparison to the corresponding undigested formula. Data are expressed as the mean ± SEM. Labeled means without a common letter differ (*p* < 0.05). **(B)** Gastric proteolysis evaluated by NH2 quantification. Formulas contained as lipids either only plant lipids (PL, *n* = 7–8), a half-half mixture of plant and dairy lipids (DL, *n* = 6–8), or a half-half mixture of plant and dairy lipids supplemented with Lf (DL+Lf, *n* = 6). SDS-PAGE, sodium dodecyl sulfate–polyacrylamide gel electrophoresis; CN, casein; β-Lg, β-lactoglobulin; α-Lac, α-lactalbumin.

Gastric protein digestion was followed by SDS-PAGE (Figure 2A). Regardless of the formula, casein was more extensively digested than β-lactoglobulin and α-lactalbumin 90 min postprandially (Figure 2A). The percentage of intact casein was lower in DL+Lf stomach compared with PL and DL, highlighting a decreased resistance of this protein to gastric digestion in the presence of DLs and probiotic Lf (Figure 2A). Intact protein percentages of β-lactoglobulin and α-lactalbumin were similar between groups. The overall gastric protein hydrolysis was higher in DL and DL+Lf piglets than in PL piglets (Figure 2B).

Overall, before and during digestion, 2,758 unique peptides (6–46 amino acids in length) were identified and derived from 19 parent proteins, of whom 8 were caseins (Supplementary Table 2). Forty to sixty peptides, mainly derived from caseins, were identified before digestion in the three infant formulas.

**Figure 3:**
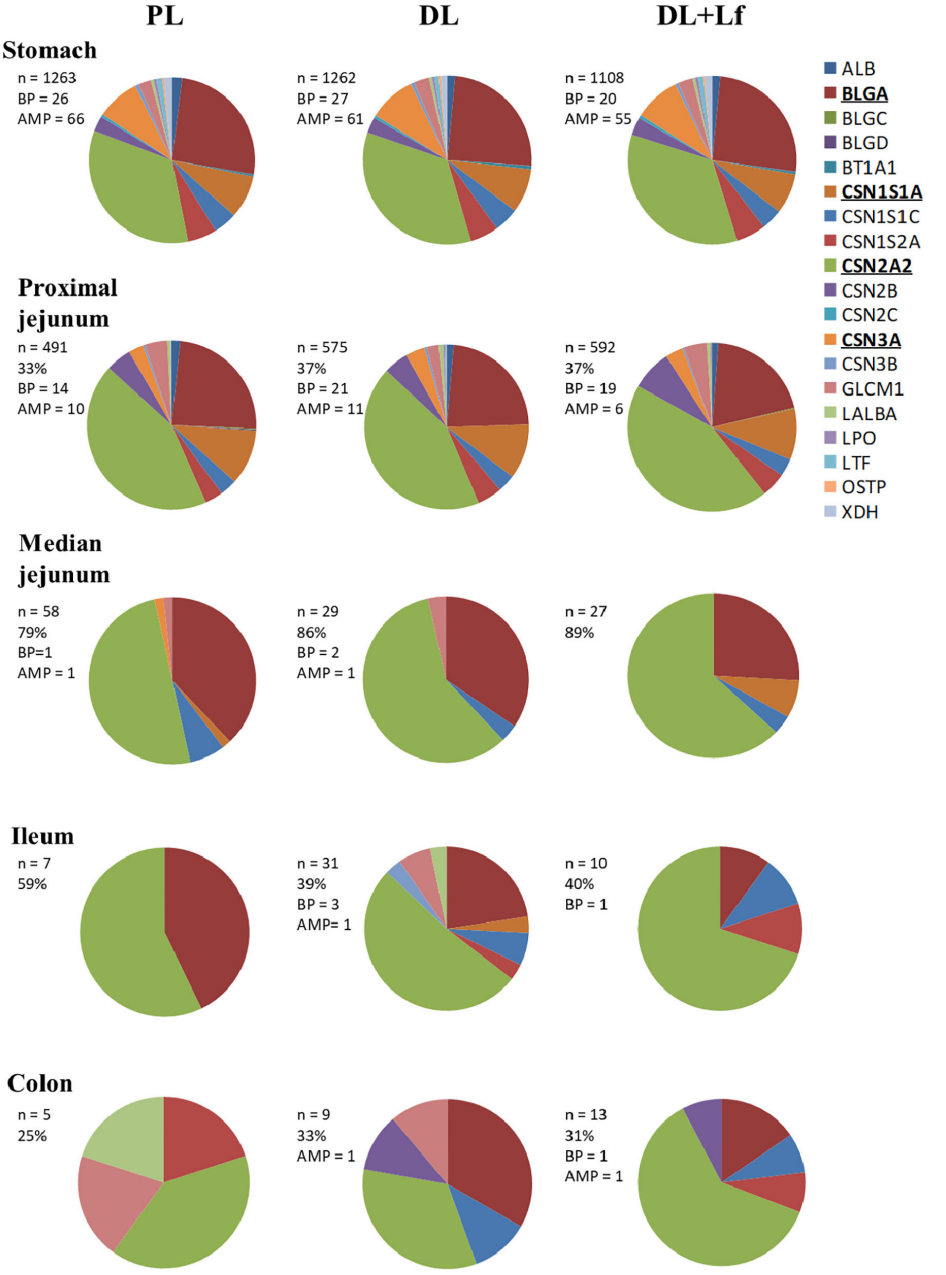
Pie chart distributions of peptides by parent proteins throughout the gastrointestinal tract. Proteins that appear in bold and underlined are the main parent proteins. In the insert, the following information are given: n, number of peptides; %, % of peptides common with the preceding compartment; BP, bioactive peptides; AMP, predicted anti-microbial peptides. Formulas contained as lipids either only plant lipids (PL, *n* = 5–8), a half-half mixture of plant and dairy lipids (DL, *n* = 5–8), or a half-half mixture of plant and dairy lipids supplemented with Lf (DL+Lf, *n* = 6–7). ALB, serum albumin; BLGA, β-lactoglobulin A; BLGC, β-lactoglobulin C; BLGD, β-lactoglobulin D; BT1A1, butyrophilin; CSN1S1A, casein α-S1 A; CSN1S1C, casein α-S1 C; CSN1S2A, casein α-S2 A; CSN2A2, β-casein A2; CSN2B, β-casein B; CSN2C, β-casein C; CSN3A, κ-casein A; CSN3B, κ-casein B; GLCM1, glycosylation-dependent cell adhesion molecule; LALBA, α-lactalbumin; LPO, lactoperoxydase; LTF, lactoferrin; OSTP, osteopontin; XDH, xanthine dehydrogenase/oxidase.

**Figure 4:**
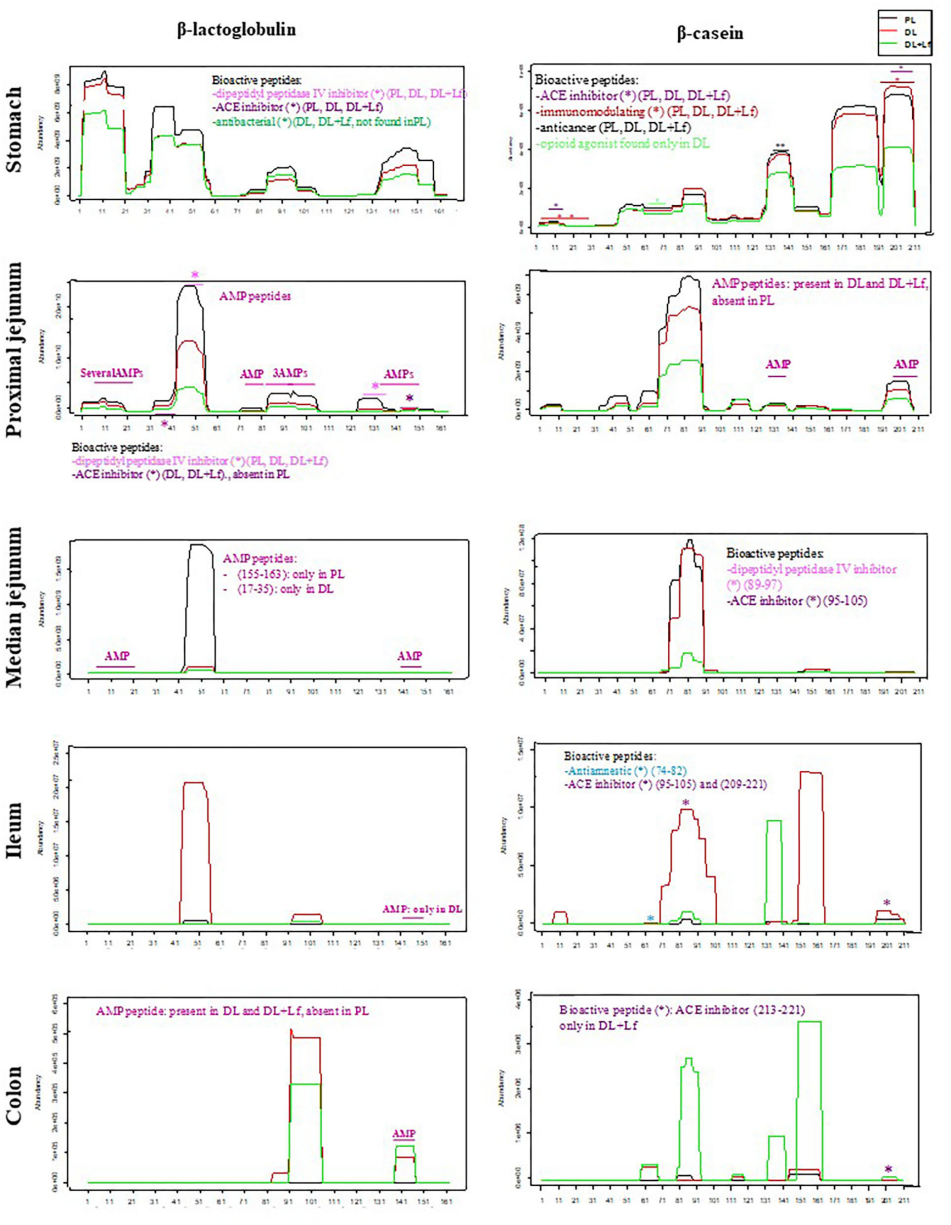
Abundance of peptides belonging to β-lactoglobulin (BLGA) and β-casein (CSN2A2) proteins and identification of bioactive peptides and AMPs throughout the gastrointestinal tract. Bioactive peptides and AMPs are identified by a star colored according to their predicted bioactivity. Formulas contained as lipids either only plant lipids (PL, *n* = 5–8), a half-half mixture of plant and dairy lipids (DL, *n* = 5–8), or a half-half mixture of plant and dairy lipids supplemented with Lf (DL+Lf, *n* = 6–7). A * indicates bioactive peptide or AMP peptides. AMP, antimicrobial peptide; ACE, angiotensin-converting enzyme.

Of the 2,758 identified peptides, 1,954 peptides (still originating from the aforementioned 19 parent proteins, Figure 3) were kept for further analyses because they were present in at least half (or half+1 in case of odd number) of the digesta samples of one of the three groups at a given site. Peptide diversity was the highest in the stomach and in proximal jejunum and largely decreased in the lowest part of the intestine (Figure 3). A large percentage of peptides were common between consecutive digestive sites, especially between proximal and the median jejunum (Figure 3). Throughout digestive sites, peptides mainly originated from β-casein (CSN2A2) and β-lactoglobulin (BLGA) (Figure 3). Distribution of peptides along these parent protein sequences was similar between groups in the stomach until the median jejunum but was more different in the ileum and the colon (Figure 4). The average peptide abundance of β-lactoglobulin and β-casein was the highest for PL and the lowest for DL+Lf from stomach to median jejunum, whereas a higher peptide abundance was observed in ileum for DL and in colon for DL+Lf especially on β-casein (Figure 4).

The number of identified bioactive peptides and AMPs was the highest in the stomach (Figure 3). Some bioactive peptides and potential AMPs were still found in proximal jejunum, but a reduced number (maximum of 4) were observed from median jejunum to the colon. Bioactive peptides originated from β-lactoglobulin, β-casein, α-s1 and α-s2 casein, and κ-casein in the stomach; from β-lactoglobulin, β-casein, α-s1 and α-s2 casein, and α-lactalbumin in the proximal jejunum; and from β-casein in the median jejunum, ileum, and colon. One peptide originated from α-s1 casein in DL+Lf ileum. Throughout the digestive sites, identified bioactivities were antibacterial, immunomodulating, dipeptidyl peptidase IV inhibitor, angiotensin-converting enzyme inhibitor, antiamnestic, binding, hamolytic, anticancer, opioid agonist, and antioxidative. Predicted AMPs originated from β-lactoglobulin, α-s1 and α-s2 casein, κ-casein, xanthine dehydrogenase/oxidase, lactoperoxidase, osteopontin, and serum albumin in the stomach; β-lactoglobulin and β-casein in proximal jejunum; and β-lactoglobulin in median jejunum, ileum, and colon. Bioactive peptides and predicted AMPs originating from the two major proteins, β-lactoglobulin and β-casein, are presented in Figure 4; for clarity, gastric AMPs are not shown.

### Effects of the Addition of Dairy Lipids and Probiotic Lf on Lipolysis

**Figure 5:**
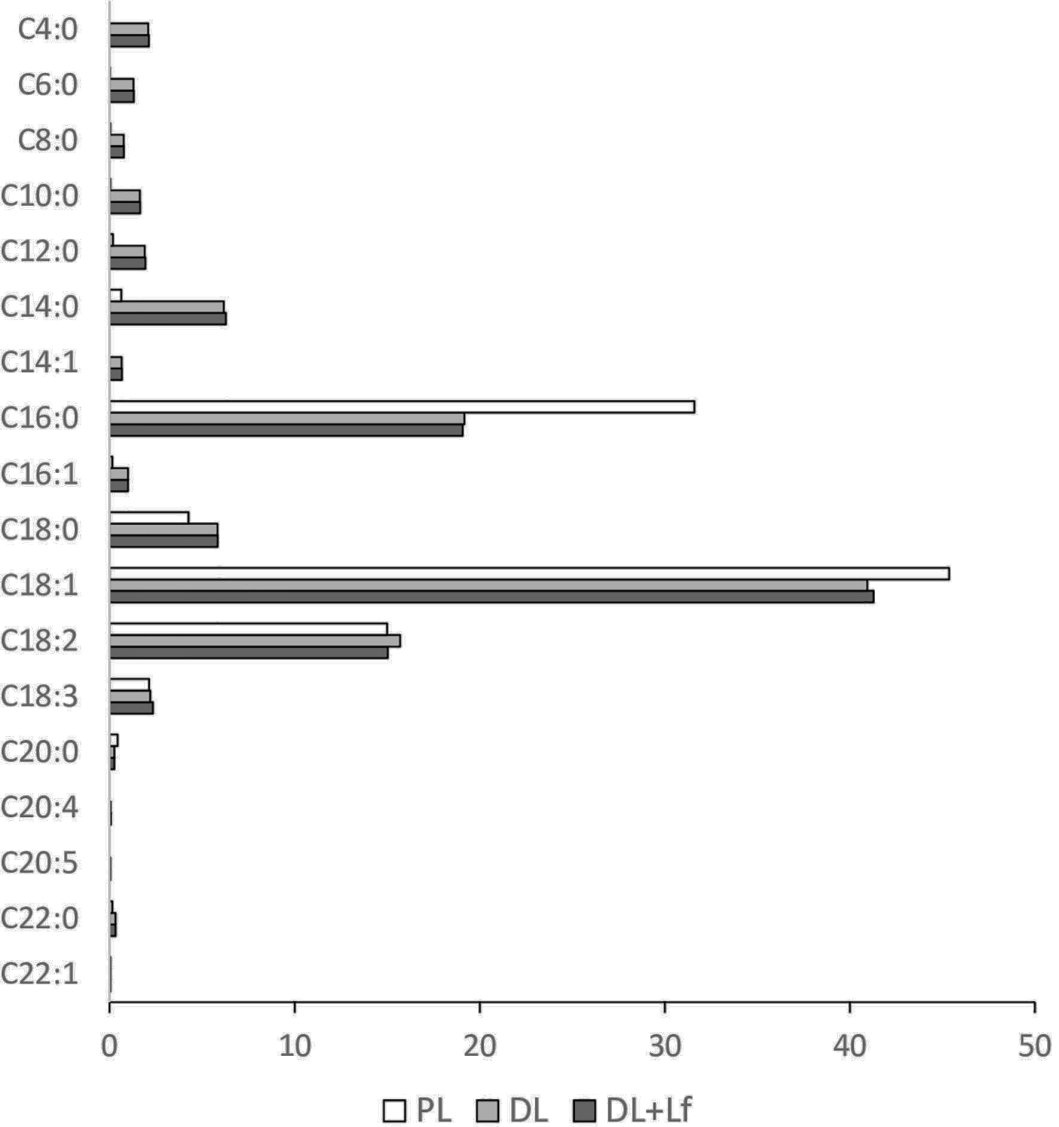
Fatty acid profile of the three infant formulas. Fatty acid concentrations are expressed in g/100 g of total fatty acids. Formulas contained as lipids either only plant lipids (PL), a half-half mixture of plant and dairy lipids (DL), or a half-half mixture of plant and dairy lipids supplemented with Lf (DL+Lf).

Fatty acid composition of the infant formulas is given in Figure 5. Except for *trans*-fatty acid content, the three infant formulas had similar content of the different classes of fatty acids (saturated and unsaturated fatty acids). However, the main individual fatty acids greatly differed among formulas, with higher levels of C18:1 and C16:0 and lower levels of C18:0 and C14:0 in PL than in DL and DL+Lf. As expected, the PL formula containing only vegetable lipids merely contains fatty acids ranging from C6:0 to C10:0, also called medium-chain fatty acids (MCFAs), and DL and DL+Lf formulas incorporating DLs as cream contained much more of these MCFAs.

**Figure 6:**
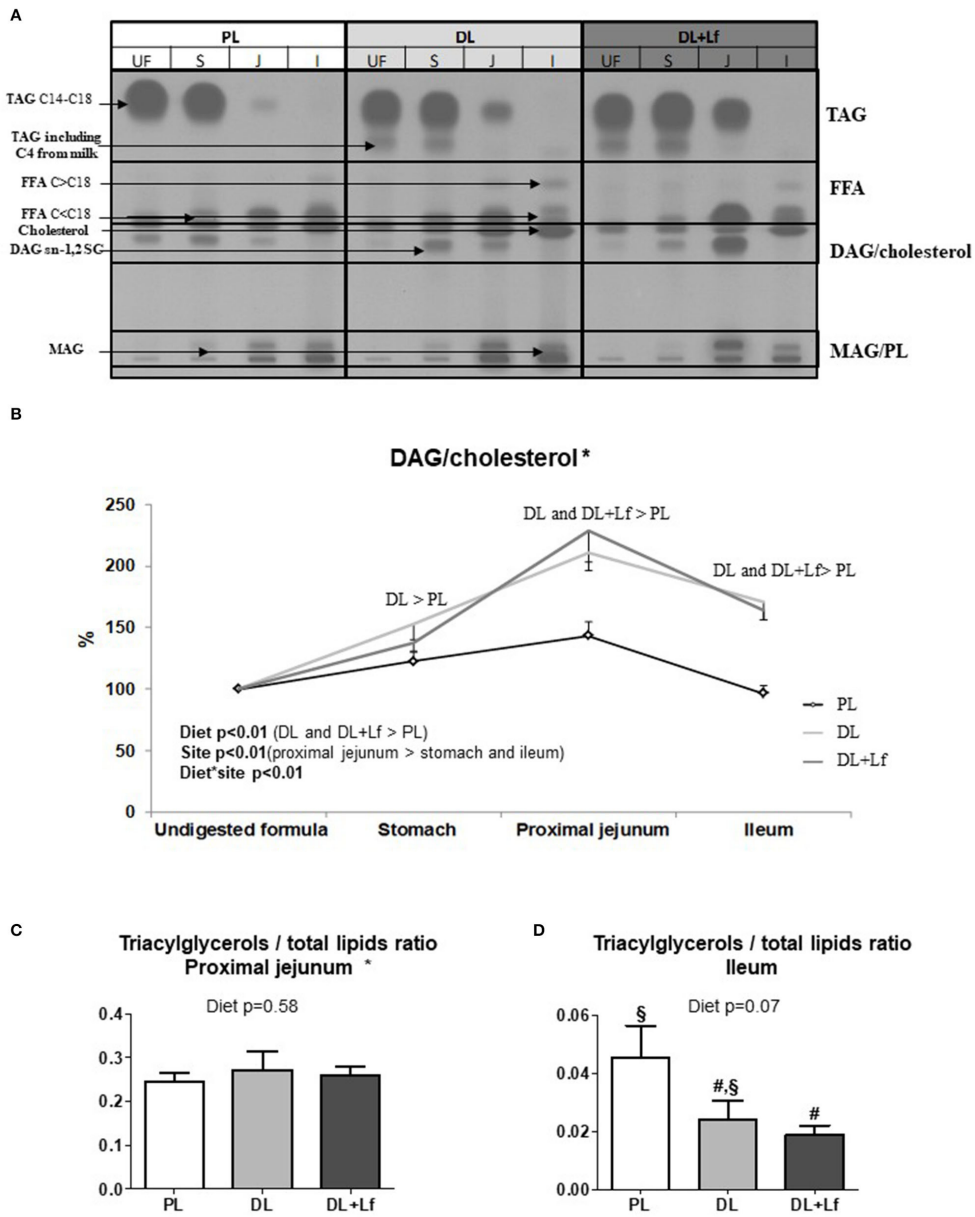
Gastric and intestinal lipolysis of PL, DL, and DL+Lf formulas. **(A)** Example of thin-layer chromatography showing the evolution of lipid classes between stomach (S), proximal jejunum (J), and ileum (I) 90 min postprandially compared to undigested formula (UF). **(B)** Longitudinal evolution of DAG/cholesterol along the intestine based on the undigested infant formula content. All written effects were significant (*p* < 0.01). **(C)** Triacylglycerols/total lipids ratio in proximal jejunum and **(D)** in ileum. Labeled bar charts without a common symbol differ (*p* < 0.1). Formulas contained as lipids either only plant lipids (PL, *n* = 7–8), a half-half mixture of plant and dairy lipids (DL, *n* = 6–8), or a half-half mixture of plant and dairy lipids supplemented with Lf (DL+Lf, *n* = 6–7). TAG, triacylglycerol; FFA, free fatty acids; SC, short chain; DAG, diacylglycerol; MAG, monoacylglycerol; PL, polar lipid. Data are expressed as the mean ± SEM. *DAG/cholesterol: *p*(diet * sex) = 0.04 (DL and DL+Lf females > PL females, DL, and DL+Lf male piglets > PL male piglets); TAG/total lipids ratio in proximal jejunum: *p*(diet * sex) = 0.02 (DL males > DL females) and *p*(sex) is not significant.

Gastric lipolysis degree was very low (<1%) and similar for all three groups. Along the digestive tract, lipolysis increased from the stomach to the ileum 90 min postprandially and was characterized by a strong decrease in triacylglycerols and an increase in lipolysis products (FFAs and diacylglycerols and monoacylglycerols) (Figure 6A). The kinetics of appearance of diacylglycerols/cholesterol were significantly impacted by the infant formula composition, depending on the intestinal site (Figure 6B). A higher percentage of diacylglycerols/cholesterol was observed in DL stomach and in DL and DL+Lf proximal jejunum and ileum compared with PL. Regardless of formula, this percentage was increased in the proximal jejunum compared with the stomach and decreased in the ileum compared with the proximal jejunum. The kinetics of appearance of FFAs and monoacylglycerols/polar lipids in small intestinal contents were not significantly different amongst formulas (data not shown). The triacylglycerols:total lipid ratio in proximal jejunum was similar between groups (Figure 6C). In contrast, it tended to decrease in DL+Lf ileum compared with PL (Figure 6D), reflecting an increased lipolysis.

### Effects of the Addition of Dairy Lipids and Probiotic Lf on Metabolism and Entero-Insular Axis

**Figure 7:**
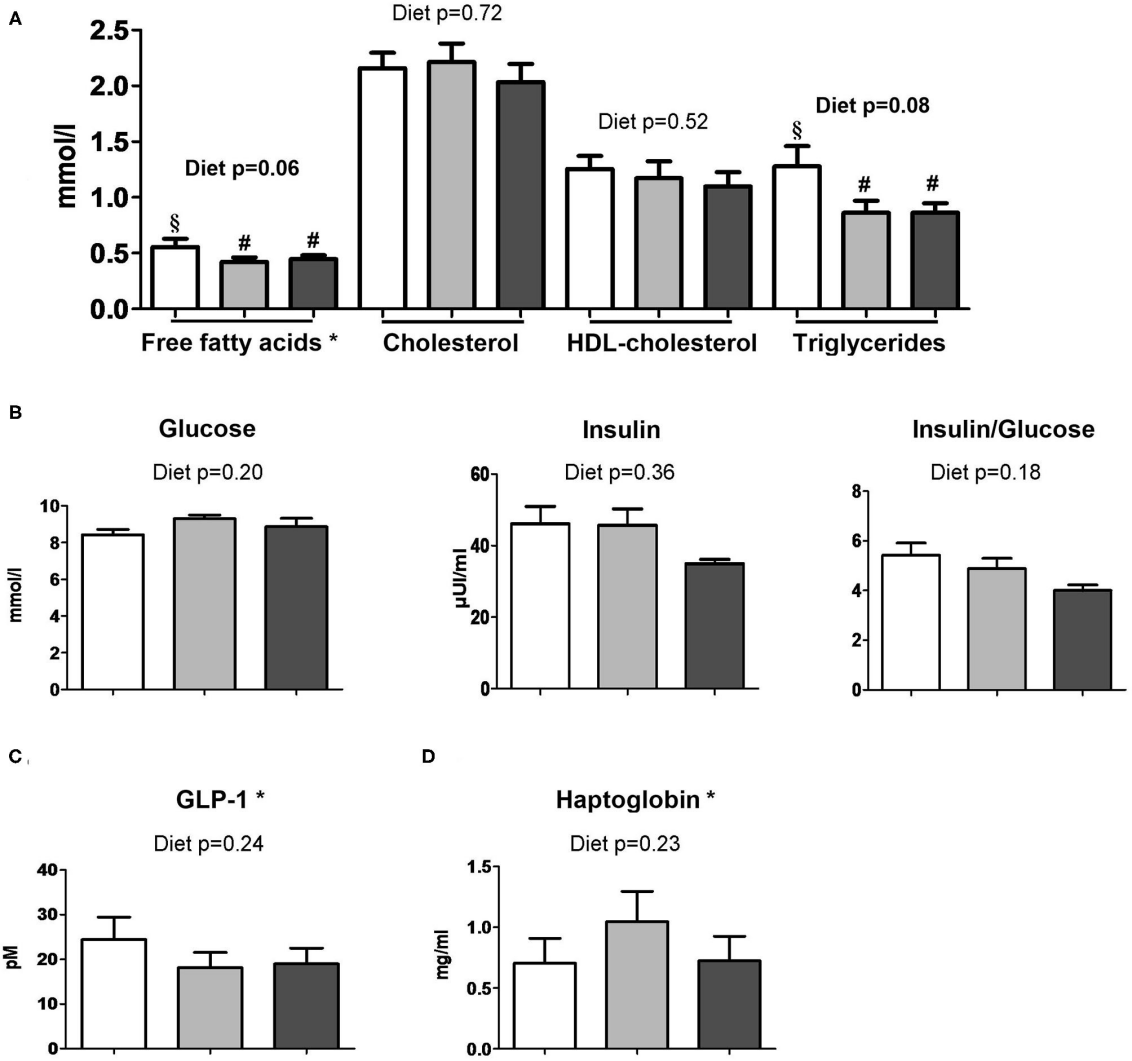
Plasma metabolic profiles of PL, DL, and DL+Lf piglets. Plasma **(A)** lipid profile, **(B)** glucose, insulin and insulin:glucose ratio, **(C)** GLP-1, and **(D)** haptoglobin concentrations 90 min postprandially. Formulas contained as lipids either only plant lipids (PL, *n* = 6–8), a half-half mixture of plant and dairy lipids (DL, 6–7), or a half-half mixture of plant and dairy lipids supplemented with Lf (DL+Lf, *n* = 6–7). Data are expressed as the mean ± SEM. Labeled means between bar graphs without a common letter or symbol differ (*p* < 0.05 and *p* < 0.1, respectively). HDL, high-density lipoprotein; GLP-1, glucagon-like peptide-1. *Free fatty acids: *p*(diet * sex) = 0.03 (PL females > DL and DL+Lf females) and *p*(sex) = 0.82; GLP-1: *p*(sex) = 0.02 (females > males); haptoglobin: *p*(diet * sex) = 0.05 (DL females > PL females) and *p*(sex) = 0.45.

**Figure 8:**
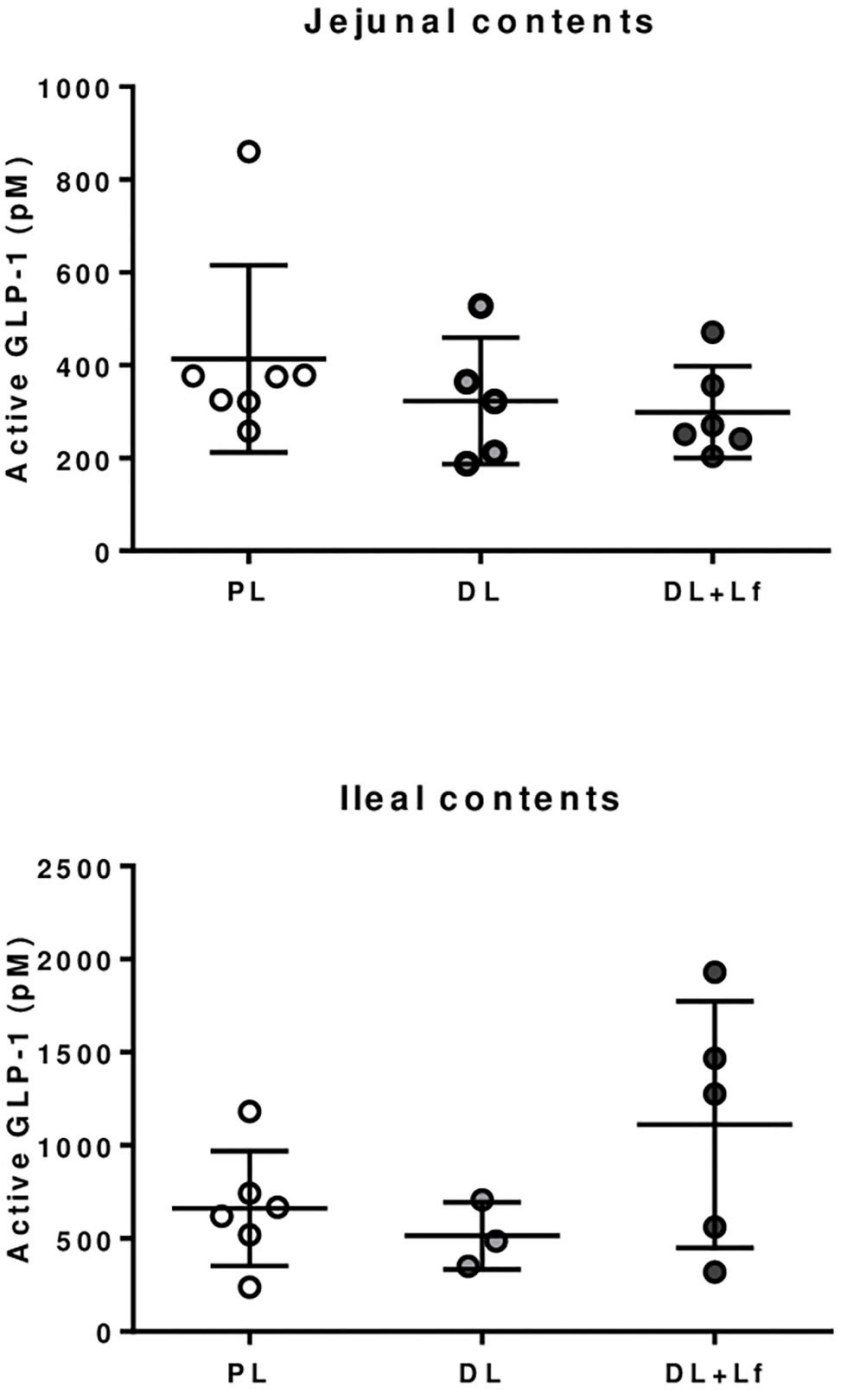
GLP-1 secretion by STC-1 endocrine cells in presence of jejunal and ileal contents of PL, DL, and DL+Lf piglets. Formulas contained as lipids either only plant lipids (PL, *n* = 6–7), a half-half mixture of plant and dairy lipids (DL, *n* = 3–5), or a half-half mixture of plant and dairy lipids supplemented with Lf (DL+Lf, *n* = 5–6).

The infant formula composition barely influenced the plasma lipid profile with tendencies to decrease plasma FFA and triglyceride concentrations in DL and DL+Lf piglets compared with PL (Figure 7A) and did not influence plasma glucose, insulin, and insulin:glucose ratio, and GLP-1 and haptoglobin concentrations (Figures 7B–D). The percentage of endocrine tissue and the number of Langerhans islets in the pancreas were decreased in DL+Lf compared with DL with no effect on the pancreas insulin content (Table 3). Mean islet diameter tended to be increased in DL compared with PL and DL+Lf (Table 3) without any difference in the pancreatic size islet distribution (data not shown). However, the infant formula composition had no effect on GLP-1 concentration and GLP-1-secreting cell number in the ileum, cecum, and colon (Supplementary Table 3). Finally, the capacity of jejunal and ileal contents to induce GLP-1 secretion by STC-1 cells did not significantly differ between formulas (Figure 8).

**Table 3:** Endocrine pancreas parameters of PL, DL, and DL+Lf piglets.

**Endocrine pancreas**	**PL**	**DL**	**DL+Lf**	**Diet effect *p* value**
				
Endocrine tissue (%)^*^	1.79 ± 0.17^a,b^	2.28 ± 0.24^b^	1.58 ± 0.07^a^	0.02
No. of islets (per 0.5-cm^2^ tissue)	250 ± 21^a,b^	291 ± 22 ^b^	221 ± 9^a^	0.05
Mean islet diameter (μm)^*^	54.6 ± 0.9^#^	56.7 ± 1.2^§^	54.7 ± 0.7^#^	0.09
Insulin content (IU/g of pancreas)	14.3 ± 0.5	15.3 ± 1.3	13.7 ± 1.0	0.58

*Data are expressed as the mean ± SEM. Labeled means in a row without a common letter or symbol differ (p < 0.05 and p < 0.1, respectively)*.

* *Endocrine tissue: p(sex) = 0.1(males > females); endocrine pancreas mean islet diameter: p(sex) < 0.01 (males > females)*.

*Formulas contained as lipids either only plant lipids (PL, n = 6–8), a half-half mixture of plant and dairy lipids (DL, n = 6), or a half-half mixture of plant and dairy lipids supplemented with Lf (DL+Lf, n = 6–7)*.

## Discussion

To our knowledge, this is the first study evaluating the impact of the addition of DLs with or without probiotic Lf in infant formulas on digestion, gut physiology, and metabolism. This study demonstrated, through the use of a set of multiscale techniques, that the addition of DLs and probiotic Lf in infant formulas affected protein and lipid digestion, decreased the endocrine tissue in the pancreas, and increased the intestine weight and the jejunal goblet cell density in Yucatan minipiglets.

A classical structure of homogenized infant formulas was observed with submicronic fat droplets in all the formulas, although most of them were in an aggregated form in the three formulas, as observed after the addition of an anionic surfactant. The observed microstructure in digesta differed alongside the digestive tract, but was not different among formulas with the present magnification. Different interfacial composition between DLs and PLs may have been present; however, interface characterization was not performed. This could be achieved after extracting and washing the lipid droplets, followed by chromatography characterization of proteins and phospholipids at the interface.

The infant formula composition affected the overall gastric proteolysis that was increased in the two groups that received DLs, potentially resulting from different interfacial composition between dairy and plant lipids. This result was in line with the lower concentration of residual intact caseins in DL+Lf stomach, but not with that in DL stomach. The former observation could be partly induced by a faster gastric emptying, as the fraction of the ingested meal remaining in stomach 90 min postprandially was the lowest in DL+Lf compared to DL and PL (24 vs. 33 and 36%, respectively), although this did not reach statistical significance. The values obtained for DL and PL were in agreement with previous results indicating that 39% of total ingested nitrogen remained in the stomach 90 min postprandially, vs. 79% 30 min after meal ingestion (16). It is unlikely that the increased proteolysis was related to a proteolytic effect of the probiotic Lf since Cardenas et al. (28) did not reveal a remarkable proteolytic activity of probiotic Lf.

Caseins, particularly β-casein, were identified in our study as the main parent protein of the peptides present in the three experimental formulas and in digestive contents. Differences in protein digestion and casein resistance previously discussed may therefore affect the release of peptides and protein/peptide biological activities. In agreement with the decreased intact casein in the DL+Lf stomach, peptides belonging to β-casein were less abundant in the stomach and proximal and median jejunum but more abundant in the colon of DL+Lf than in that of PL and DL. This result is in accordance with a faster gastric emptying induced by dairy lipids and probiotic Lf. Different bioactive peptides were observed along the sequence of β-casein and β-lactoglobulin. Particularly, an inhibitor of the DPP-IV was found to be less abundant for DL + Lf than for the other formulas from the stomach until the median jejunum. The DDP-IV is produced all along the intestine, and significant DPP-IV–like activity occurs in the microbiota (29). This peptidase is involved in the last step of dietary protein digestion, with the specificity of being able to hydrolyze peptides containing proline, unlike pancreatic proteases. Thus, the reduction of DPP-IV activity may alter protein digestion and absorption (29). Besides, DPP-IV is known to inactivate two incretins, GLP-1 and GIP, involved in the control of glucose metabolism. In the present study, such effect was not apparent as plasma GLP-1 contents were similar among groups. This can be explained by the fact that the DPP-IV activity in the bloodstream is mainly that of hematopoietic and endothelial cells.

Different distributions of peptide origin were observed between groups from median jejunum to colon. Especially, PL had lower peptide diversity in the ileum and the colon compared to DL and DL+Lf, highlighting a specific effect of dairy lipids on proteolysis. These different peptides may differentially modulate gut microbiota and gut physiology, or on the other hand, dairy lipids and probiotic Lf may have differentially modulated gut physiology and consequently its digestive capacity. Particularly, the present study has demonstrated a beneficial effect of probiotic Lf on non-specific host defenses, increasing goblet cell density in jejunum, in agreement with Lf-induced greater MUC-2 expression observed *in vitro* in HT29 cells (30). It should be noted that the intestinal transit time may also have been changed by specific peptides released during casein digestion such as the opioid ones (31, 32).

Another important result was that the infant formula composition modulated lipid digestion. This was not apparent at the stomach level, where lipolysis appeared to be low; however, this may have been underestimated because of the gastric emptying of the FFAs released before digesta collection. The percentage of diacylglycerols/cholesterol present at 90 min postprandially was higher for DL and DL+Lf than for PL in proximal jejunum and ileum, which is likely due to a higher abundance of diacylglycerols sn-1,2 from DL and DL+Lf compared with PL. Concomitantly, tendencies to decreased plasma FFA and triglyceride concentrations in DL and DL+Lf piglets compared with PL were observed. This could be the result of a different metabolic fate of the lipolysis products and/or a reduced and/or slower lipid absorption with dairy lipids. Differences observed among groups in terms of lipolysis may therefore trigger health effect. In addition, the nature of the fatty acid released can also play different luminal and systemic functions. Our previous study on milk fat and MFGM fragments in piglets showed a significant increase of the mucosal immune system maturation and modification of the fecal microbiota composition (8). Particularly, the sphingosine and MCFAs present in dairy lipids are known for their antimicrobial activity and their modulation of gut microbiota establishment with increased *Lactobacillus* and *Bifidobacterium* (33). Contrary to what was expected, no difference in plasma cholesterol was observed between groups. It is noteworthy that the cholesterol content of the experimental formulas containing dairy lipids was lower than that of sow milk [0.80 and 10.7 mg for 100 mL of PL and DL (±Lf) formulas, respectively, vs. 145 mg for 100 mL of sow milk (34)].

Overall metabolism was not affected by the infant formula composition. However, our results highlighted that probiotic Lf decreased the percentage of endocrine pancreas and the number of Langerhans islets. Similar effect of “pancreatic savings” was observed in piglets supplemented with prebiotic short chain fructooligosaccharides (scFOS), suggesting a lower insulin demand and potentially a better insulin sensitivity of peripheral tissues to insulin (35) even if the postprandial insulin and the insulin/glucose ratio did not display a significant decrease in DL+Lf. As supplementation of both probiotic Lf and prebiotic scFOS is susceptible to modify intestinal microbiota, this “pancreatic savings” could be related to intestinal microbiota modification between groups. Intestinal GLP-1 secreting function (i.e., number and percentage of GLP-1 secreting enteroendocrine L-cells) and postprandial GLP-1 concentration were not different between groups. This was in agreement with the *in vitro* STC-1 results demonstrating no difference in the GLP-1 secretion stimulating capacity of intestinal contents, despite differences in their peptide and lipid compositions. In contrast, we reported a long-term promoting effect of Lf on GLP-1 secreting function in the adult minipigs under a hyperenergetic-diet challenge, suggesting that a deleterious nutritional environment was necessary to reveal the metabolic programming (11). Finally, a trophic effect of probiotic Lf was observed on duodenum and jejunum weights, as well as on small intestine length. This is coherent with a recent study that also observed an intestinal trophic effect of this specific strain (36). Mechanisms responsible for the modulation of intestinal growth by Lf remain unknown, but could involve, for instance, a modulation of microbiota composition (11).

The use of piglets as models for infants was a strength of our study, the piglet having many common features with infant regarding nutritional physiology and functional gut maturation (7). Furthermore, regarding lipid digestion, as for breast milk, palmitic acid in sow milk is mainly on the sn-2 position 70%, (37), and as for humans, pig pancreatic lipase mainly hydrolyzes fatty acids on the sn-1,3 positions, and palmitic acid is well-absorbed through the gut epithelium as a monoacyl glycerol (38). Therefore, piglets are very good models for infants in this regard. However, our study also displayed some limitations. For instance, it would have been optimal to have a fourth group of animals receiving the PL formula plus probiotic. However, because of economical and ethical constraints, we had to limit the number of experimental piglets and could consider only three groups. In particular, we selected the three groups PL, DL, and DL+Lf to achieve our goal of investigating the role of re-introduction of dairy lipids in infant formula on its digestion and on the intestinal physiology, and its eventual synergy with probiotic Lf. Also, a basal (fasting) point would be needed to evaluate the amplitude of the postprandial (90 min) response and compare it between formulas. However, as infants, piglets eat every 2 h during the suckling period, so there is technically no fasting period that could have been taken as a reference. The timepoint 90 min was therefore a good compromise for assessing the digestion process in suckled minipigs (16).

In conclusion, our data provide interesting knowledge about how infant formulas with different lipid nature (plant vs. dairy) and probiotic content may be differently digested, with consequences on gut physiology. More precisely, the addition of dairy lipids in infant formula modulated the digestion of lipids, whereas the addition of Lf increased proteolysis, had an intestinal trophic effect, increased the number of goblet cells, and induced a “pancreas savings” effect. Our results corroborate the synergistic properties of MFGMs and probiotics reported on mucosal B- and T-cell proliferation and mucosal IgA-secreting cells (39). The adhesion of lactic acid bacteria strains to MFGM, previously demonstrated for *Lactobacillus reuteri*, may participate in the greater impact of the combination of dairy lipids and Lf compared to individual ingredients (40). All these effects had potential nutritional relevance due to bioactive peptides and AMPs and lipolysis products, and displayed beneficial, although moderate, effects on non-specific host defenses and intestinal size, as well as metabolism. Overall, we did not notice any effect of dairy lipids with or without Lf on piglet growth. This was also the case in infants receiving formulas supplemented with either MFGM or another probiotic strain (*Lactobacillus paracasei* ssp. *paracasei* strain) (41).

## Data Availability Statement

The original contributions presented in the study are included in the article/Supplementary Material, further inquiries can be directed to the corresponding author/s.

## Ethics Statement

The animal study was reviewed and approved by The ethics committees of CREEA (Rennes Committee of Ethics in Animal Experimentation) and of the France's Ministry of Higher Education and Research approved the entire protocol (authorization #2016011111546978).

## Author Contributions

IL, SB, DD, PL, and CB conceived the project and ML, IL, SB, DD, PL, and CB designed the experiment. IC, PL, and CB provided the infant formulas. ML, OM, AC, IN, VB-B, BC, AD, IL, and SB performed analysis. ML, OM, BC, AD, DD, SB, and IL analyzed the data. ML, AD, SB, and IL wrote the draft manuscript. All authors reviewed and approved the final manuscript.

## Conflict of Interest

IC, PL, and CB are employees of Lactalis. ML received a grant from Lactalis Recherche et Développement (Retiers, France) and from the Association Nationale de la Recherche et de la Technologie (ANRT) (Grant CIFRE No. 2014/0580). The remaining authors declare that the research was conducted in the absence of any commercial or financial relationships that could be construed as a potential conflict of interest.
