# Supplementary material for: Addition of Dairy Lipids and Probiotic Lactobacillus fermentum in Infant Formulas Modulates Proteolysis and Lipolysis With Moderate Consequences on Gut Physiology and Metabolism in Yucatan Piglets
Source: Front Nutr. 2021 Feb 24;8:615248. doi: 10.3389/fnut.2021.615248 (PMC7943452; doi:10.3389/fnut.2021.615248)
Supplement: Supplementary file 1 [file Table_1.DOCX]

# Supplement Table 1. Tissue weights and intestinal morphometry of PL, DL and DL+Lf pigs at euthanasia (28 days of age)

|  | **PL (n=6-9)** | **DL (n=6-9)** | **DL+Lf (n=6-8)** | **Diet effect p-value** |
| --- | --- | --- | --- | --- |
| **Body weight** (kg) ***** | 3.17 ± 0.19 | 3.29 ± 0.30 | 2.86 ± 0.14 | 0.54 |
| **Brain** (g/kg BW) ***** | 15.8 ± 0.7 | 15.3 ± 1.3 | 17.6 ± 0.9 | 0.50 |
| **Liver** (g/kg BW) ***** | 36.2 ± 0.6 ^a^ | 37.2 ± 0.7 ^b^ | 37.8 ± 1.2 ^a,b^ | **0.04** |
| **Pancreas** (g/kg BW) | 1.57 ± 0.09 | 1.42 ± 0.11 | 1.61 ± 0.08 | 0.32 |
| **Perirenal adipose tissue** (g/kg BW) | 8.40 ± 0.70 | 8.94 ± 0.65 | 7.48 ± 0.47 | 0.26 |
| **Subcutaneous adipose tissue** (g/kg BW) * | 5.27 ± 0.26 | 5.65 ± 0.50 | 4.43 ± 0.29 | 0.18 |
| **Empty stomach** (g/kg BW) | 7.08 ± 0.18 | 6.75 ± 0.40 | 6.92 ± 0.25 | 0.73 |
| **Gastric content** (g/kg BW) | 13.2 ± 1.8 | 11.9 ± 1.7 | 10.3 ± 1.7 | 0.50 |
| **Empty duodenum** (g/kg BW) | 1.95 ± 0.15^#,§^ | 1.90 ± 0.16 ^#^ | 2.41 ± 0.19 ^§^ | **0.09** |
| **Duodenal content** (g/kg BW) | 13.2 ± 1.8 | 11.9 ± 1.7 | 10.3 ± 1.7 | 0.50 |
| **Duodenal length** (m/kg BW) | 0.05 ± 0.01 | 0.06 ± 0.01 | 0.07 ± 0.01 | 0.23 |
| **Empty proximal jejunum** (g/kg BW) | 28.5 ± 3.4 | 28.5 ± 2.2 | 27.9 ± 1.5 | 0.60 |
| **Proximal jejunal content** (g/kg BW) ***** | 3.63 ± 1.20 | 3.44 ± 0.57 | 2.93 ± 1.05 | 0.77 |
| **Proximal jejunal mucosa density** (g/cm) | 0.20 ± 0.01 | 0.21 ± 0.02 | 0.23 ± 0.01 | 0.51 |
| **Proximal jejunal villi length** (µm) | 570 ± 42 | 567 ± 25 | 533 ± 16 | 0.65 |
| **Proximal jejunal villi surface** (µm^2^) * | 68513 ± 5075 | 70524 ± 2292 | 67417 ± 4343 | 0.32 |
| **Proximal jejunal villi goblet cell number** (/villus) | 8.40 ± 1.35 ^a^ | 10.0 ± 1.4 ^a,b^ | 14.0 ± 1.1 ^b^ | **0.02** |
| **Proximal jejunal crypt depth** (µm) | 298 ± 12 | 295 ± 15 | 316 ± 12 | 0.50 |
| **Proximal jejunal crypt surface** (µm^2^) * | 13122 ± 738 | 12513 ± 802 | 14647 ± 945 | 0.20 |
| **Proximal jejunal crypt goblet cell number** (/crypt) | 13.6 ± 0.9 | 14.9 ± 1.5 | 16.7 ± 1.2 | 0.20 |
| **Median jejunal content** (g/kg BW) | 1.98 ± 0.32 | 1.60 ± 0.26 | 2.67 ± 0.55 | 0.25 |
| **Empty median jejunum** (g/kg BW) | 12.1 ± 1.7 ^a^ | 11.1 ± 0.9 ^a^ | 16.3 ± 2.1 ^b^ | **0.02** |
| **Ileum length** (m/kg BW) * | 0.54 ± 0.13 | 0.43 ± 0.07 | 0.48 ± 0.04 | 0.64 |
| **Ileal content** (g/kg BW) | 3.30 ± 0.91 | 1.24 ± 0.39 | 2.08 ± 0.41 | 0.13 |
| **Empty ileum** (g/kg BW) | 14.9 ± 2.7 | 12.8 ± 1.7 | 13.6 ± 1.8 | 0.69 |
| **Ileal mucosa density** (g/cm) | 0.14 ± 0.01 | 0.14 ± 0.01 | 0.13 ± 0.01 | 0.83 |
| **Ileal villi length** (µm) | 425 ± 31 | 415 ± 25 | 368 ± 34 | 0.31 |
| **Ileal villi surface** (µm^2^) | 47341 ±  3872 ^b^ | 46162 ± 2812 ^a,b^ | 37795± 3899 ^a^ | **0.09** |
| **Ileal villi goblet cell number** (/villus) | 12.0 ± 1.6 | 11.7 ± 1.2 | 9.1 ± 1.5 | 0.32 |
| **Ileal crypt depth** (µm) | 215 ± 9 | 219 ± 18 | 227 ± 15 | 0.84 |
| **Ileal crypt surface** (µm^2^) | 10116 ± 516 | 10482 ± 1429 | 11545 ± 1228 | 0.63 |
| **Ileal crypt goblet cell number** (/crypt) | 10.9 ± 1.1 | 11.4 ± 0.5 | 11.5 ± 0.8 | 0.88 |
| **Total small intestine length** (m/kg BW) * | 2.00 ± 0.09 ^a,b^ | 1.87 ± 0.15 ^a^ | 2.26 ± 0.07 ^b^ | **0.08** |
| **Empty caecum** (g/kg BW) | 2.37 ± 0.12 | 2.18 ± 0.16 | 2.39 ± 0.20 | 0.36 |
| **Caecal content** (g/kg BW) * | 2.85 ± 0.29 | 2.20 ± 0.50 | 3.37 ± 0.74 | 0.22 |
| **Caecal crypt depth** (µm) | 389 ± 30 | 366 ± 17 | 369 ± 22 | 0.75 |
| **Caecal crypt surface** (µm^2^) | 26305 ± 3106 | 24385 ± 1632 | 24914 ± 2015 | 0.83 |
| **Caecal goblet cell number** (/crypt) | 27.4 ± 3.2 | 24.5 ± 1.9 | 26.2 ± 3.0 | 0.42 |
| **Colon length** (m/kg BW) * | 0.32 ± 0.02 | 0.33 ± 0.02 | 0.35 ± 0.01 | 0.81 |
| **Empty colon** (g/kg BW) | 15.8 ± 1.1 | 15.2 ± 1.0 | 16.0 ± 1.1 | 0.87 |
| **Colonic content** (g/kg BW) | 5.12 ± 0.71 | 6.28 ± 0.37 | 5.93 ± 0.58 | 0.36 |
| **Colonic goblet cell number** (/crypt) | 36.5 ± 4.5 | 42.6 ± 2.4 | 38.1 ± 4.3 | 0.48 |
| **Total intestine length** (m/kg BW) * | 2.32 ± 0.10 | 2.20 ± 0.17 | 2.61 ± 0.08 | 0.11 |

Formulas contained as lipids either: only plant lipids (PL), a half-half mixture of plant and dairy lipids (DL) or a half-half mixture of plant and dairy lipids supplemented with Lf (DL+Lf). BW, body weight. Data are expressed as the mean ± SEM.

* Body weight: p(sex)=0.06 (males > females); brain: p(sex)=0.08 (females > males); liver: p(sex)=0.03 (but not significant post-hoc) and p(diet*sex)=0.05 (DL+Lf males > PL females); subcutaneous adipose tissue: p(sex)=0.04 (males > females); proximal jejunal content: p(diet*sex)=0.02 but not significant post-hoc and p(sex)=0.24; jejunal villi area/surface: p(sex)=0.03 and p(diet*sex)=0.01 (PL female piglets < DL female piglets (0.079), PL female piglets < PL male piglets (0.065); ileum length: p(sex)=0.03 (females > males); small intestine length: p(sex)=0.02 (females > males); caecal content: p(sex)=0.02 (females > males); colon length: p(sex)=0.02 (females > males); intestine length: p(sex)=0.02 (females > males).

# Supplement Table 2: Identification of peptides parent proteins

Nb AA, number of amino acids of the protein (without the peptide signal).

**Supplement Table 3. Intestinal GLP-1-secreting function in PL, DL and DL+Lf pigs**

|  | **PL (n=6-8)** | **DL (n=6-7)** | **DL+Lf (n=5-7)** | **Diet effect**  **p-value** |
| --- | --- | --- | --- | --- |
| **Ileal mucosa GLP-1** (pmol/ g tissue) * | 213 ± 26 | 211 ± 39 | 177 ± 37 | 0.32 |
| **Caecal GLP-1** (pmol/ g tissue) | 59.0 ± 6.5 | 73.1 ± 13.5 | 74.6 ± 15.8 | 0.74 |
| **Colonic GLP-1** (pmol/ g tissue) | 70.5 ± 5.8 | 81.1 ± 10.2 | 80.3 ± 12.9 | 0.82 |
| **Caecal GLP-1-secreting cells number** (n/mm^2^) | 5.80 ± 0.99 | 6.09 ± 0.74 | 6.08 ± 1.34 | 0.96 |
| **% caecal GLP-1-secreting cells / total enteroendocrine cells** (%) | 25.2 ± 2.8 | 22.2 ± 3.8 | 24.3 ± 1.5 | 0.76 |
| **Colonic GLP-1-secreting cells number** (n/mm^2^) | 8.62 ±1.05 | 8.12 ± 0.88 | 9.80 ± 1.30 | 0.47 |
| **% colonic GLP-1-secreting cells / total enteroendocrine cells** (%) | 26.2 ± 1.5 | 25.3 ± 3.2 | 24.4 ± 1.9 | 0.86 |

Formulas contained as lipids either: only plant lipids (PL), a half-half mixture of plant and dairy lipids (DL) or a half-half mixture of plant and dairy lipids supplemented with Lf (DL+Lf). Data are expressed as the mean ± SEM.

* Ileal mucosa GLP-1 (pmol/ g tissue): p(sex)=0.03 (females > males).

# Supplement Figure 1. Piglet growth. (A) Weight gain, (B) average energy intake per day, and (C) food efficiency between PND3 and PND26 for PL (n=9), DL (n=9) and DL+Lf pigs (n=8).

Formulas contained as lipids either: only plant lipids (PL), a half-half mixture of plant and dairy lipids (DL) or a half-half mixture of plant and dairy lipids supplemented with Lf (DL+Lf). BW, body weight. Data are expressed as the mean ± SEM.
